# Frequency of sarcopenia and associated factors among hospitalized elderly patients

**DOI:** 10.1186/s12891-015-0570-x

**Published:** 2015-05-06

**Authors:** Bruno Prata Martinez, Anne Karine Menezes Santos Batista, Isabela Barboza Gomes, Flávia Milholo Olivieri, Fernanda Warken Rosa Camelier, Aquiles Assunção Camelier

**Affiliations:** Bahia School of Medicine and Public Health, Av. Dom João VI, n° 275, Brotas, Salvador, Bahia Brazil; State University of Bahia, Salvador, Bahia Brazil; City Hospital, Salvador, Bahia Brazil

**Keywords:** Sarcopenia, Elderly, Hospital, Mass muscle, Grip strength

## Abstract

**Background:**

Sarcopenia is an important public health problem that affects mainly elders, and has negative consequences, such as disability and even death. Due to the lack of studies evaluating sarcopenia in elderly persons hospitalized in Brazil, the aim of the present study was to describe the frequency of sarcopenia and associated factors among elders in a hospital in the city of Salvador - Brazil.

**Methods:**

This cross-sectional study included 110 hospitalized elderly patients in a multi-specialty hospital in Salvador-BA, Brazil. Inclusion criteria: were elders aged ≥60 years between the first and fifth day of hospitalization; who were able to walk without external assistance; with medical permission to walk, and who did not take vasoactive and inotropic drugs. The diagnosis of sarcopenia was determined by combining the reduction in skeletal muscle mass with muscle weakness (women, <20 kg; men, <30 kg) and/or poor physical performance (gait speed ≤0.8 m/s). To obtain reduced skeletal muscle mass, the skeletal muscle mass index ≤6.37 kg/m^2^ for women and ≤8.90 kg/m^2^ for men was used. Cognitive function, Charlson index, admission profile (clinical and surgical), smoking, falls suffered in the last year and physical inactivity prior to admission were also evaluated. The frequency of sarcopenia was described in percentages with their respective confidence intervals and logistic regression was performed for multivariate analysis of factors associated with sarcopenia.

**Results:**

Among the 110 patients included, the frequency of sarcopenia was 21.8%, with 10.0% being of the severe type. There was a predominance of clinical profile (59.1%), such as heart disease (20.0%), pneumonia (13.6%) and skin infections (9.1%), with a Charlson index of 5.4 ± 1.8. The factors associated with sarcopenia were age (OR = 1.14; 95% CI = 1.06 to 1.23), clinical profile on admission (OR = 5.15; 95% CI = 1.16–22.9) and smoking (OR = 7.8; 95% CI = 1.53–39.9).

**Conclusions:**

The frequency of sarcopenia in elderly hospitalized patients was high (1 in 5 elderly) and anthropometric equation can be a viable and inexpensive alternative to screening and programming intervention in this population.

## Background

The reduction in muscle mass that occurs with aging was initially described by Rosenberg in 1989 [1]. In 1998, Baumgartner and colleagues developed a practical method to assess sarcopenia, using the muscle mass index (MMI). These authors suggested that the accepted values for sarcopenia were two standard deviations below the standard values for a specific population as a function of gender and age [2]. However, the current definition of sarcopenia, established by the European Working Group on Sarcopenia in Older People (EWGSOP), includes a reduction in strength and/or physical performance, in addition to a reduction in skeletal muscle mass [3].

Older people are highly susceptible to sarcopenia, which maybe be associated with increased muscle weakness [4], falls/fractures [5], limitations in activities [6,7] and increased risk of death [8-10]. However, the weakness may be related to other causes, such as neural and muscle factors, and not the reduction in mass only [3,11]. Previous studies have reported that the global frequency of sarcopenia ranges from 3.0% to 36.1% among community-dwelling older people [2,12-16]. This wide variation can be explained by the different methods and diagnostic criteria used. The use of anthropometric measures is a simple and inexpensive methods to assess sarcopenia, but with lower accuracy. Nonetheless, Lee et al. [17] developed predictive equations for muscle mass based on anthropometric measurements, and verified that these measurements were strongly correlated with data obtained by the use of magnetic resonance imaging [17] and dual-energy X-ray absorptiometry (DEXA) [14].

Most studies about sarcopenia included community-dwelling elderly and few studies have reported frequency of the disease among the hospitalized elderly. Hospitalization may have negative consequences such cognitive impairment, physical disability, prolonged hospitalization, social isolation and decreased quality of life [18]. Therefore, early identification of sarcopenia is essential, particularly in the hospital setting, considering that some risk factors are present. Due to the lack of studies evaluating sarcopenia in elderly hospitalized patients in Brazil, the aim of the present study was to describe the frequency of sarcopenia and associated factors among elderly patients in a hospital in the city of Salvador - Brazil.

## Methods

This study included 110 elderly patients admitted to the City hospital, a multi-specialty hospital providing care for patients from both the public and supplementary health systems, located in the city of Salvador, Bahia, Brazil. The study was conducted in the period from August 2013 to January 2014. The inclusion criteria were hospitalized individuals aged ≥60 years, examined in the time between the first and fifth day of hospitalization, who were not under treatment with vasoactive and inotropic drugs, able to walk without external assistance or auxiliary devices, who had medical permission to walk, who had no pain, dyspnea or cardiopulmonary change that prevented them from performing physical activity.

The primary variables were anthropometric measurements, handgrip strength, gait speed, cognitive function, history of falls in the last year and smoking status. The secondary variables obtained were medical admission diagnosis, admission profile (clinical or surgical), length of stay hospital during data collection, and the Charlson index to assess comorbidities. On a daily basis, the researchers checked the electronic medical record system to find patients who met the criteria for inclusion in the study. The Research Ethics Committee of the Bahia School of Medicine and Public Health approved the project under Protocol Number 336.469 / 2013. After being duly informed about the research, all patients signed a term of free and informed consent to participate in the study.

### Measurement

For the diagnosis of sarcopenia, muscle mass, handgrip strength and physical performance were measured. Skeletal muscle mass was estimated (SMM) using the Lee equation [17] for patients with BMI <30: (0.244 * body weight) + (7.8 * height) + (6.6 * gender) − (0.098 * age) + (race − 3.3); with body weight in kilograms and height in meters. The value 0 must be used for women, 1 for men, then 0 for whites, 1.4 for blacks and −1.2 for Asians [17]. A recent Brazilian study demonstrated strong agreement between DEXA and this predictive equation for muscle mass (k = 0.74; p <0.001), with a high specificity (89%) and sensitivity (86%) [14]. For elderly patients with BMI ≥30 kg/m^2^, the specific anthropometric equation [17]: {height * (0.007444 * CAG^2^ + 0.00088 * CTG^2^ + 0.00441 * CCG^2^) + 2.4 * gender – 0.048 * age + race + 7.8} was used.

The skinfold thickness measurements (S) in the arm, thigh and medial part of the calf were performed by trained evaluators; and the circumferences of the limbs (C_limb_) in the mid upper arm, mid thigh and mid calf were also measured to the nearest 1 mm, according to anthropometric standardization [19]. We used the *Lange* caliper (USA) to measure the skinfold thickness. Three measurements were performed and the mean of the measurements was obtained for analysis. To remove the fat component, the corrected value of the circumference (C_m_: Climb - π.S) was obtained [17]. Subsequently, the SSM was divided by height squared to obtain the skeletal muscle mass index (MMI). The criteria used to assess the reduction in skeletal muscle mass were values ≤6.37 kg/m^2^ for female patients and ≤8.90 kg/m^2^ for male patients, which are equivalent to 20% of lowest percentile distribution reported by Alexandre et al. [16], according to the studies by Newman et al. [20] and Delmonico et al. [21].

Body mass index (BMI) was also calculated by dividing the weight (in kg) by the square of height (in m). The values established by the Lipschitz et al. [22] recommendation, which allows for changes in body composition owing to aging, were used to classify the following: underweight, BMI <22 kg/m^2^; normal weight, BMI between 22 and 27 kg/m^2^ and excess weight, BMI >27 kg/m^2^ [22].

To assess handgrip strength, the participants were asked to sit on a chair with elbows positioned at a 90° angle and exert maximum force, using a *Saehan* hydraulic dynamometer (Saehan Corporation, 973, Yangdeok-Dong, Masan 630–728, Korea). This dynamometer presented high reliability in comparison with the gold standard, which is the Jamar dynamometer [23]. This measurement was performed three times, with a 1-min rest interval between measurements, and the highest values were considered. For assessing muscle weakness, values <20 kg and <30 kg were considered for female and males, respectively [24].

The parameter used to evaluate physical performance was the 6-m gait speed test. For this purpose, participants were asked to walk a distance of 10 m on a flat surface, in a straight line, as fast as they could, and the time taken to walk the middle 6 m was measured. The highest values were considered, and values ≤0.8 m/s indicated poor physical performance [25].

Cognitive function was assessed using the mini-mental state examination (MMSE), which quantifies various domains of cognition, with a score ranging from 0 to 30 [26]. The report of low physical activity pre-admission was graded positive for elderly people who were inactive or who performed physical activity <2 times a week [27]. To evaluate the severity of the patients’ comorbidities, data were collected by means of the Charlson comorbidity index within the first 24 hours of admission [28]. The elderly who reported having smoked at least one cigarette per day in the last month were considered smokers [29]. Self-reports of falls in the past year were also evaluated.

### Statistical analysis

The numerical variables were expressed as means and standard deviations, and the categorical data were expressed in percentages with their respective confidence intervals. The association between sarcopenia and length of stay at the time of data collection, and the Charlson index were analyzed using the chi-square test (Length of hospital stay during data collection: ≤3 days and 3–5 days, Charlson index: ≤4 and ≥5). The intergroup comparisons of the variables age, BMI, Charlson index, cognitive function, handgrip strength, and gait speed were performed using the Student’s *t*-test for independent variables. Multivariate analysis of factors associated with sarcopenia was performed by the logistic regression (backward method), which included the six most significant variables: age, cognitive function, admission profile (clinical or surgical), smoking, age ≥80 years and reports of physical inactivity pre-hospitalization (less than 2x per week). One hundred patients were evaluated, considering an estimated error of 7%, a significance level of 5%, and an expected rate of sarcopenia of 15%, based on previous studies [15,16,30]. The analyses were performed using the SPSS software version 14.0, and p-values of <0.05 were considered significant.

## Results

**Figure 1:**
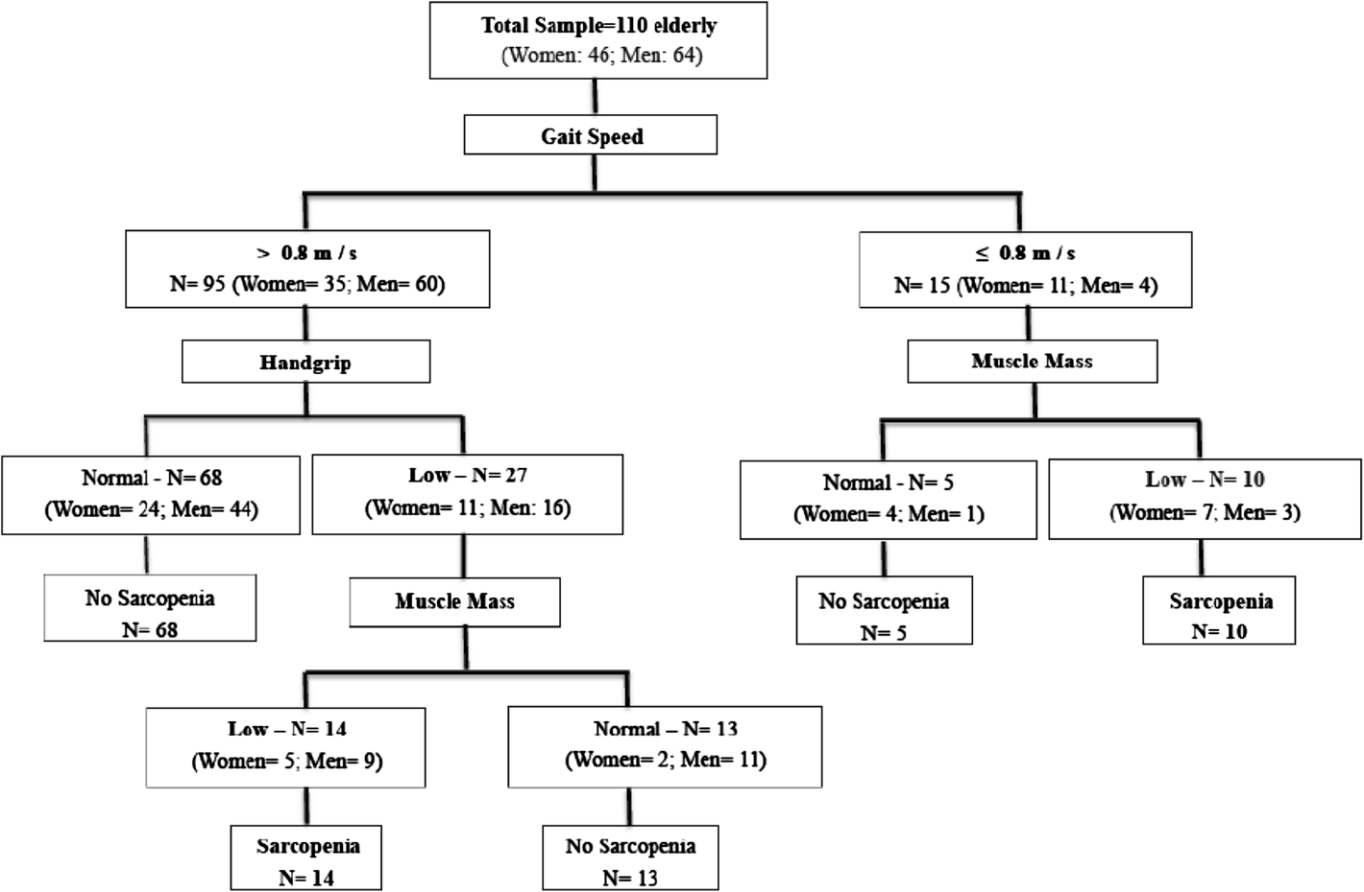
Algorithm of 110 elderly patients evaluated by the study according European working group on Sarcopenia in older people.

Descriptive data of 110 elderly patients evaluated in this study are described in Table 1 and Figure 1. According to the definitions of sarcopenia related by the European Working Group on Sarcopenia in Older People, the prevalence of sarcopenia was 21.8% (95% CI = 14.5–30.7). Of the 24 older people with sarcopenia, 41.7% had severe sarcopenia. Ten elderly patients (9.1%) had pre-sarcopenia, since they had only a reduction in skeletal muscle mass.

**Table 1:** **Descriptive characteristics of the sample of 110 hospitalized elderly patients included in the study, categorized in elderly with sarcopenia (sarcopenia and severe sarcopenia) and without sarcopenia**

	**Sarcopenia**		**No sarcopenia**	
**Variables**	**% (n)**	**Mean/SD**	**% (n)**	**Mean/SD**
**Age**		78.9 ± 9.5		68.8 ± 6.8
60–69 years	16.7 (4)		62.8 (54)	
70–79 years	33.3 (8)		27.9 (24)	
≥80 years	50.0 (12)		9.3 (8)	
**BMI**		21.5 ± 2.4		26.5 ± 4.6
Underweight	54.2 (13)		10.5 (9)	
Normal weight	445.8 (11)		53.5 (46)	
Excess weight	0.0 (0)		36.0 (31)	
**Gender**				
Male	50.0 (12)		60.5 (52)	
Female	50.0 (12)		39.5 (34)	
**Admission profile**				
Clinical	87.5 (21)		51.2 (44)	
Surgical	12.5 (3)		48.8 (42)	
**MMSE**		20.4 ± 5.7		24.7 ± 4.4
**Charlson index**		6.3 ± 1.9		5.2 ± 1.7
≤4	25.0 (6)		39.5 (34)	
≥5	75.0 (18)		60.5 (52)	
**Length of stay**				
**(days)**		2.8 ± 1.7		2.7 ± 1.6
1–3	62.5 (15)		65.1 (56)	
≥3	37.5 (9)		34.9 (30)	
**Smoking**				
Absent	70.8 (17)		93.0 (80)	
Present	29.2 (7)		7.0 (6)	
**Physical activity**				
**<twice a week**				
No	17.4 (4)		41.6 (32)	
Yes	82.6 (19)		58.4 (45)	
**Falls in the last year**				
Absent	62.5 (15)		77.9 (67)	
Present	37.5 (9)		22.1 (19)	
**SMM** (kg)		17.5 ± 4.1		23.1 ± 5.1
**MMI** (kg / m^2^)		6.8 ± 1.6		8.8 ± 1.7
**Handgrip** (kg)		18.9 ± 6.7		30.5 ± 8.4
**Gait speed** (m / s)		0.95 ± 0.4		1.36 ± 0.41

BMI = Body mass index; MMSE = Mini mental state exam; SMM = Skeletal muscle mass; MMI = Muscle mass index.

In this study, there was a predominance of clinical patients (59.1%), and the most common reasons for admission were abdominal surgeries (34.5%), heart disease (20.0%), pneumonia (13.6%) and skin infections (9.1%). Length of hospital stay during data collection was 2.7 ± 1.6 days and 64.5% of measurements were obtained between the first and third days of admission. There was no difference in the occurrence of sarcopenia among the different length of stay during data collection. Sixty-four percent of participants reported practicing <2 days of physical activity per week before hospital admission. With regard to BMI, normal weight (51.8%) predominated, followed excess weight (28.2%) with 20.0% being classified as underweight.

In the intergroup comparison, elderly patients with sarcopenia exhibited a lower level of cognitive function than the group without sarcopenia, as well as higher values for age and Charlson index (Table 2). In the comparison of categorical variables the highest frequencies of sarcopenia occurred in the clinical profile on admission, older age groups, smokers, those with worse cognitive function and in those who reported low levels of physical activity pre-hospitalization (Table 2). After multivariate analysis, only age, admission clinical profile and the presence of smoking remained associated with the presence of sarcopenia in the sample of hospitalized elderly patients.

**Table 2:** **Univariate and multivariate analysis of factors associated with sarcopenia in the sample of 110 hospitalized elderly**

	**Univariate analysis**		**Multivariate analysis**	
**Variables**	**OR (CI 95%)**	**P-value**	**OR (CI 95%)**	**P-value**
**Age**	1.16 (1.09–1.24)	0.001	1.14 (1.06–1.23)	0.001
**Age (60–69 years)**	0.12 (0.04–0.38)	0.001		
**Age (70–79 years)**	1.29 (0.49–3.4)	0.606		
**Age (≥80 years)**	9.8 (3.3–28.8)	0.001		
**Gender (Male)**	0.7 (0.3–1.6)	0.36		
**Admission profile (Clinical)**	6.7 (1.9–24.1)	0.001	5.15 (1.16–22.9)	0.031
**MMSE**	0.87 (0.77–0.93)	0.001		
**Charlson index**	1.4 (1.07–1.82)	0.013		
**Charlson index (≥5)**	2.3 (0.8–6.9)	0.11		
**Length of stay (days)**	1.1 (0.4–2.9)	0.81		
**Smoking (Present)**	5.5 (1.6–18.4)	0.007	7.8 (1.5–39.9)	0.014
**Falls in the last year (Present)**	2.1 (0.8–5.6)	0.13		
**Physical activity prior (less than 2x per week)**	3.4 (1.1–10.9)	0.034		

## Discussion

### Frequency of sarcopenia

The frequency of sarcopenia among hospitalized elderly patients was 21.8% and higher than the results of a recent study involving older people with acute disease (10%) [31]. The main explanation for the lower frequency in the reported study is the different method used to determine muscle mass, which consisted of calculating the arm circumference and triceps skinfold, differently from the present study, in which the anthropometric equation was used. In addition, the previous study did not assess physical performance for diagnosing sarcopenia, which may have underestimated its occurrence.

In a recent study conducted in Brazil, Alexander et al. [16] also adopted the Lee anthropometric equation to assess muscle mass, and reported a prevalence of 15.4% in community-dwelling older people, which was slightly lower than the present study results. Although these were not hospitalized elderly, they had a high frequency of comorbidities, including hypertension (61%), osteoarthritis (32.4%), and heart disease (20.8%).

Similar to our study, Yamada et al. [32] reported 21.8% prevalence of sarcopenia in community-dwelling older people in Japan, based on the use of electrical bioimpedance measurement of skeletal muscle mass. This rate was higher than that of older people in UK (7.8%) [15] and of those aged >80 years in the study of Belfrail (12.5%) [33]. Differently from the study involving British older people, which primarily used anthropometric skinfold thickness measurements for the definition of decreased muscle mass [15], whereas we adopted the Lee anthropometric equation in our study. Despite the lower accuracy of anthropometric measurements to predict muscle mass in comparison with the gold standard [14-16], recent studies have used anthropometry because of its low cost and operational simplicity for the early identification of sarcopenia, which may be considered a valid option in daily care for screening patients in need of specific interventions.

Despite its negative outcome, such as disability [7] and increased mortality [10], sarcopenia remains understudied, and limited knowledge is available about hospitalized older people. Quantifying the prevalence of sarcopenia is essential to warn healthcare professionals about this disease and its negative consequences, including death [31,10]. Furthermore, hospitalization usually is associated with diseases and comorbidities, which can trigger sarcopenia through the increase in inflammatory response, physical inactivity and malnutrition [34,35]. The elderly patients included in the study were not submitted to specific strength or balance training before the measurements taken for the present study.

### Factors associated with sarcopenia

The higher frequency of sarcopenia in older age groups found in this study is similar to the findings of previous studies [2,15,16] that have reported a rise in frequency, mainly among those in age groups older than 80 years. A justification for this is the possible reduction in motor neurons, which was demonstrated by McNeil et al. [36] who observed a reduction of almost 50% in motor neurons between 60–80 years of age. In the present study each year of age from 60 years onwards, there was a 14% increase in the odds ratio of the elderly having sarcopenia. In this study, half of the elderly patients with sarcopenia were older than 80 years, but after multivariate analysis this was not statistically significant, probably due to the small sample size.

In addition to the predominance of patients with clinical profile on admission in the sample, 87.5% older people with sarcopenia exhibited the clinical profile, and the odds ratio for patients with clinical profile to have sarcopenia was 5.15 compared with patients with surgical profile, after multivariate analysis. One explanation for this finding is that most of the surgeries were of the elective abdominal type (34.5%) and patients with a clinical profile were older and had a longer stay in hospital during data collection. This may have influenced the low frequency of cases of sarcopenia in surgical patients, considering the lack of significant differences in the Charlson index between the surgical and medical patients (5.1 ± 1.7 vs. 5.7 ± 1.9, respectively; p-value = 0.35). Another aspect is that patients with a clinical profile had a predominance of heart disease, pneumonia and infections, suggesting the influence of triggering inflammatory mechanism of protein degradation, malnutrition and consequent sarcopenia [34,35]. Another factor associated with sarcopenia was smoking, which in this study showed an odds ratio of 7.8. This can be explained by the possible increase in the inflammatory response triggered by smoking [37], and other causes such as impairment of energy supply and oxygen to the muscle and metabolic pathways, promoted by reduced blood flow [16,38,39].

In the intergroup comparison of sarcopenia, BMI values were lower in subjects with sarcopenia, however the average value was classified as normal and similar to those of recent studies [15,33,35] and to the Brazilian study [16]. Only 20.0% of the 110 older patients studied were classified as underweight, and this result differs from that of another study involving hospitalized older people, in which 62% of the participants were underweight [31].

With regard to cognitive functions, no significant association was found after multivariate analysis, corroborating the study of Kan et al. [40] who also found no association after adjustment for confounding variables. Therefore, longitudinal studies with larger sample sizes are warranted to better assess the correlations, causal effects, and the long-term effects of sarcopenia.

The study had some limitations, such as the cross-sectional nature of the study, which prevents a cause-effect relationship in some relations. Nonetheless, the use of high-precision instruments would have prevented the development of the present study, considering the operational and financial issues involved. The frequency of sarcopenia may also have been underestimated, because older people with more severe and acute conditions and those with inability to perform physical tests and/or who used an auxiliary device or external assistance were excluded. In addition, the anthropometric equation applied in the twelve elderly with BMI ≥30 led to a higher risk of bias in the measurement of skeletal muscle mass, which cannot indicate sarcopenic obesity in the elderly. Another limitation was the failure to use a special tool to evaluate the nutritional aspect, in addition to BMI.

## Conclusions

The frequency of sarcopenia among hospitalized elderly patients was high (1 in 5 elderly), which demonstrates the need for further research into its causes and consequences in hospitalized patients. The use of anthropometric measurements may be practical and feasible for the early detection of sarcopenia and should be used in the hospital setting because of the negative outcomes of this condition, and the possibility of managing certain modifiable factors. The factors associated with sarcopenia in elderly hospitalized patients were age, clinical profile on admission and smoking.

## Abbreviations

CTG: Corrected thigh girth
CCG: Corrected calf girth
CAG: Corrected arm girth
Kg: Kilogram
M / S: Meters per second
MMI: Skeletal muscle mass index
Kg / m^2^: Kilogram/meter^2^
K: Kappa
OR: Odds ratio
CI: Confidence interval
DEXA: Dual-energy X-ray absorptiometry
ASM: Appendicular skeletal muscle mass
BMI: Body mass index
MMSE: Mini-mental state examination
SPSS: Statistical Package for the Social Science
